# The association between retina thinning and hippocampal atrophy in Alzheimer’s disease and mild cognitive impairment: a meta-analysis and systematic review

**DOI:** 10.3389/fnagi.2023.1232941

**Published:** 2023-08-23

**Authors:** Shuntai Chen, Dian Zhang, Honggang Zheng, Tianyu Cao, Kun Xia, Mingwan Su, Qinggang Meng

**Affiliations:** ^1^School of Traditional Chinese Medicine, Beijing University of Chinese Medicine, Beijing, China; ^2^Department of Neurology, Dongzhimen Hospital, Beijing University of Chinese Medicine, Beijing, China; ^3^Department of Oncology, Guang’anmen Hospital, China Academy of Chinese Medical Sciences, Beijing, China; ^4^Department of Respiratory, Guang’anmen Hospital, China Academy of Chinese Medical Sciences, Beijing, China

**Keywords:** Alzheimer’s disease, OCT, hippocampus, retina, MRI

## Abstract

**Introduction:**

The retina is the “window” of the central nervous system. Previous studies discovered that retinal thickness degenerates through the pathological process of the Alzheimer’s disease (AD) continuum. Hippocampal atrophy is one of the typical clinical features and diagnostic criteria of AD. Former studies have described retinal thinning in normal aging subjects and AD patients, yet the association between retinal thickness and hippocampal atrophy in AD is unclear. The optical coherence tomography (OCT) technique has access the non-invasive to retinal images and magnetic resonance imaging can outline the volume of the hippocampus. Thus, we aim to quantify the correlation between these two parameters to identify whether the retina can be a new biomarker for early AD detection.

**Methods:**

We systematically searched the PubMed, Embase, and Web of Science databases from inception to May 2023 for studies investigating the correlation between retinal thickness and hippocampal volume. The Newcastle-Ottawa Quality Assessment Scale (NOS) was used to assess the study quality. Pooled correlation coefficient *r* values were combined after Fisher’s Z transformation. Moderator effects were detected through subgroup analysis and the meta-regression method.

**Results:**

Of the 1,596 citations initially identified, we excluded 1,062 studies after screening the titles and abstract (animal models, *n* = 99; irrelevant literature, *n* = 963). Twelve studies met the inclusion criteria, among which three studies were excluded due to unextractable data. Nine studies were eligible for this meta-analysis. A positive moderate correlation between the retinal thickness was discovered in all participants of with AD, mild cognitive impairment (MCI), and normal controls (NC) (*r* = 0.3469, 95% CI: 0.2490–0.4377, *I*^2^ = 5.0%), which was significantly higher than that of the AD group (*r* = 0.1209, 95% CI:0.0905–0.1510, *I*^2^ = 0.0%) (*p* < 0.05). Among different layers, the peripapillary retinal nerve fiber layer (pRNFL) indicated a moderate positive correlation with hippocampal volume (*r* = 0.1209, 95% CI:0.0905–0.1510, *I*^2^ = 0.0%). The retinal pigmented epithelium (RPE) was also positively correlated [*r* = 0.1421, 95% CI:(−0.0447–0.3192), *I*^2^ = 84.1%]. The retinal layers and participants were the main overall heterogeneity sources. Correlation in the bilateral hemisphere did not show a significant difference.

**Conclusion:**

The correlation between RNFL thickness and hippocampal volume is more predominant in both NC and AD groups than other layers. Whole retinal thickness is positively correlated to hippocampal volume not only in AD continuum, especially in MCI, but also in NC.

**Systematic review registration:**

https://www.crd.york.ac.uk/PROSPERO/, CRD42022328088.

## Introduction

Alzheimer’s disease (AD) is a progressive, insidious onset neurodegenerative disease that accounts for 60∼80% of dementia (Alzheimer’s Association, 2020), and which affects more than 50 million people worldwide. No treatment has been proven to be effective for AD and it has caused a global burden on economies and health care systems (Berk et al., 2014; den Haan et al., 2018b). The insidious onset of the disease brings potential challenges for the treatment of this progressive disease. In the National Institute on Aging and Alzheimer’s Association Research Framework, the definition of the disease has shifted from syndromal to a biological construct which focuses on the biomarkers grouped as β-amyloid deposition (A), pathologic tau (T), and neurodegeneration (N) [AT(N)] (Jack et al., 2018). The traditional approach to detecting *in vivo* biomarkers appears relatively late when clinical symptoms arise, and has high-cost and invasiveness limitations. Thus, effective interventions for early detection may significantly reduce underdiagnosis and give patients more time by postponing the progression of AD.

Mild cognitive impairment (MCI) is an intermediate stage between normal aging and dementia (Thompson et al., 2004; Ridha et al., 2008; Jack et al., 2009; Morra et al., 2009; Albert et al., 2011). The annual conversion rate from MCI to AD is approximately 10% ∼15%. On average, most MCI patients convert to AD within 5 years from the first diagnosis (Tábuas-Pereira et al., 2016). AD is characterized by memory decline, and neurobiological changes in the medial temporal lobe can occur years before memory decline appears. The earliest brain atrophy in Alzheimer’s disease typically initiates through the hippocampal pathway. The atrophy rate of the medial temporal lobe, including the hippocampus, and the whole brain (Fox et al., 1999; Sluimer et al., 2010) correlates closely with the progression of neurodegeneration. Hippocampal atrophy has been proven to be a valid structural biomarker (Dubois et al., 2007) of AD diagnosis with the simplest means of assessment and is more sensitive than Aβ deposition to changes from MCI to moderate AD (Jack et al., 2009; Frisoni et al., 2010; Sluimer et al., 2010).

The retina originates from the diencephalon during embryonic development (London et al., 2013) and is considered to be the “window” of the central nervous system (CNS) as they share similar neurobiology in neuronal cells and microvasculature. For this reason, the retina is now considered a promising biomarker for AD diagnosis as it might mirror similar pathology as neurodegenerative diseases (Cheung et al., 2017). Previous studies have discovered a multitude of amyloid β (Aβ) deposits in the nerve fiber layer (NFL) and ganglion cell layer (GCL) in AD patients (Löffler et al., 1995; Leger et al., 2011; Koronyo et al., 2017; Grimaldi et al., 2019), which could be associated with neuronal loss in the retinal GCL, inner nuclear layer (INL), and outer nuclear layer (ONL). Increased Aβ deposits (Koronyo et al., 2017; den Haan et al., 2018b) and phosphorylated tau (pTau) diffusion spreading in the inner layer of retina AD patients has been observed in post-mortem retinal slices, as well as aggregation of Aβ 2.7 times more than that of age-matched controls (Grimaldi et al., 2019). Optical coherence tomography (OCT) is a non-invasive, comparatively low-cost retinal imaging technology. Changes of OCT parameters in AD have been documented in recent years. Cross-sectional studies have shown that the peripapillary retinal nerve fiber layer (pRFNL), RNFL in the superior and inferior quadrants, the macular ganglion cell layer-inner plexiform layer (mGCL-IPL), and macular thicknesses are significantly decreased in people with preclinical Alzheimer’s disease or mild cognitive impairment (Jindahra et al., 2010; Larrosa et al., 2014; Cunha et al., 2016, 2017; den Haan et al., 2017; Chan et al., 2019; Katsimpris et al., 2022). Additionally, several researchers investigated the correlation between retinal thickness and hippocampal atrophy through retinal OCT inspection and structural MRI (Rotenstreich et al., 2019; Shi et al., 2020; Sergott et al., 2021). Previous meta-analyses have identified associations between retinal measurements of OCT and AD and MCI patients, reflecting degenerated retinal thickness compared to healthy controls (Chan et al., 2019; Ge et al., 2021). However, the association between biomarkers of the retina and hippocampal atrophy has not yet been systematically studied. This meta-analysis and systematic review aimed to address whether retinal thickness changes coordinate hippocampal atrophy during the progression of cognition decline in MCI and AD. It is also hoped to discuss under which circumstances the quantified correlation may be prominent and the potential reasons behind it.

## Methods

This study was conducted following the Preferred Reporting Items for Systematic Reviews and Meta-Analysis (PRISMA) guidelines (Moher et al., 2010). The protocol of this review was registered at PROSPERO (PROSPERO Registration Number: CRD42022328088).

### Search strategy

The electronic databases of PubMed, Embase, and the Wed of Science were searched from the establishment of the databases to May 2023. Searching strategies were structured as in Supplementary File 1. Two researchers (SC and DZ) separately implemented an online search and supplemented it with references from the relevant studies. Abstracts were screened and full articles read to preclude irrelevant studies and additional citations.

### Study selection

Studies were eligible if: (1) studies were cohort studies and/or cross-sectional studies on living humans; (2) participants were clinically diagnosed with AD, MCI, or were healthy controls; (3) the thickness parameters of the retinal images were assessed through OCT and hippocampal volume measurement from structural MRI images could be acquired from both hemispheres or unilaterally; (4) studies had reported the correlation coefficient using either the Pearson or Spearman’s method; (5) studies were peer-reviewed studies published in the English language. Any disagreements were resolved through the discussion.

### Quality assessment

The methodological quality was assessed based on the Newcastle-Ottawa Quality Assessment Scale (NOS) (Wells et al., 2000) by two independent researchers (MS and DZ). MS assessed the quality of the individual studies and DZ checked independently. The score was scaled from 0 to 9, with scores over 7 points considered high quality. No primary studies were scored under 7 based on the assessment. Disagreements were resolved through discussion and with a third reviewer when necessary.

### Data extraction

Data were extracted on participants’ characteristics, measurements of the retina and hippocampal structure, cognition assessment, and the correlation coefficient *r* between retinal thickness and hippocampal volume. All coefficients *r* were extracted when statistically significant. In studies where the coefficient correlation was absent, SPSS 26.0 software (IBM, Inc., Chicago, IL, USA) was used to run the correlation coefficient *r* from the original data.

### Analysis

The Pearson correlation measures the strength between two variables, while the linear regression model describes a linear relationship between two variables. These two coefficients cannot be combined technically. Thus, in this study, we chose the effect sizes to be the Pearson correlation coefficients. Correlation coefficients *r* were synthesized in RStudio (the “meta” package, 1.3.959).^1^ Studies were combined when the correlation coefficients r were reported in at least two studies. In most cases, the r value does not approach bivariate normal distribution and cannot be directly synthesized since the variance depends on the sample size and population parameters. However, through correction of the Fisher estimator (Berry and Mielke, 2000), bias from these sample correlations could only be partially eliminated. Therefore, we introduce this Fisher’s z transformation to convert the correlation coefficient r to obtain a basic distribution. We fit the r value from each study in the equation z’ = 0.5[ln(1 + *r*)–ln(1–*r*)] and obtained the z value. Then, the syntheses of z were pooled in the meta-analysis.

Random effect models were performed to pool the z value. Egger’s test was used to detect publication bias. Heterogeneity was assessed using the *I*^2^ statistic. We did not implement a funnel plot because the number of studies was less than 10. We implemented subgroup analysis and meta-regression to investigate the potential sources of heterogeneity according to the following categories: (1) participants of AD/MCI/NC; (2) left/right/both side(s); (3) retinal measurement sites: the retinal nerve fiber layer (RNFL), ganglion cell layer (GCL), retinal pigmented epithelium (RPE), inner nuclear layer (INL), and inner plexiform layer (IPL).

## Results

### Study characteristics

**FIGURE 1:**
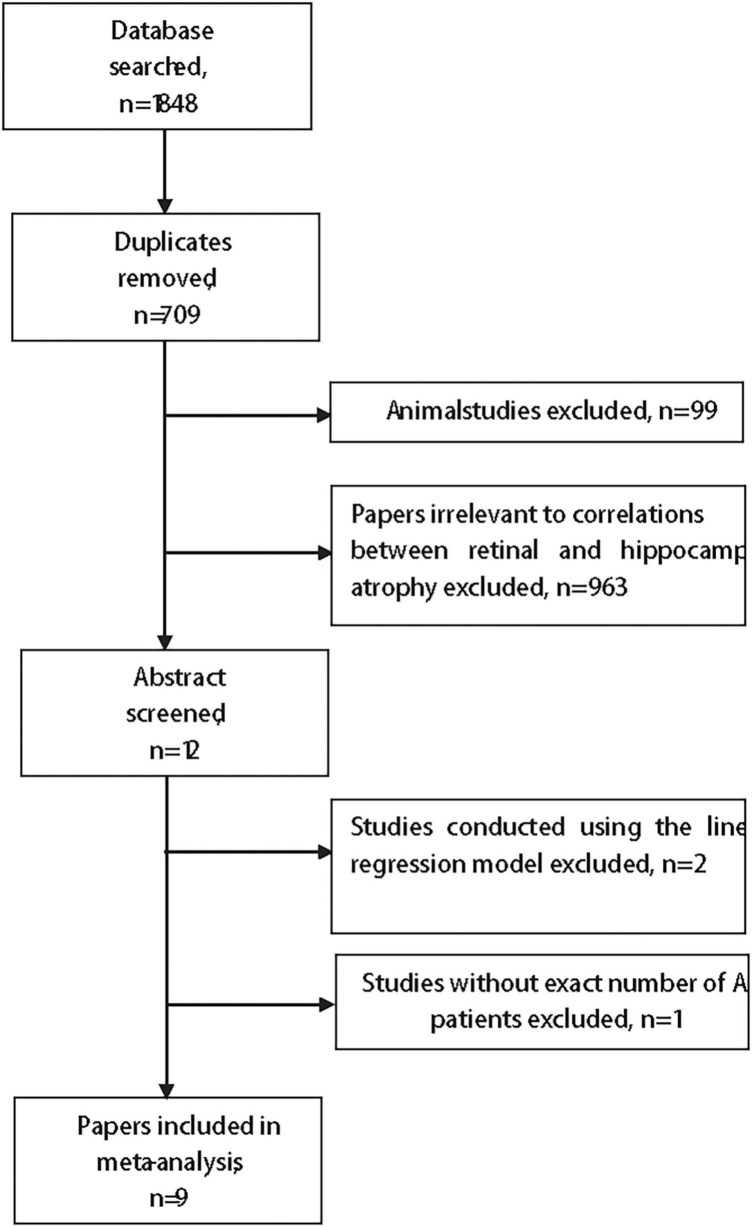
The flow chart of the search strategy.

The searches initially identified 1,139 unique citations (Figure 1). After screening the titles and abstract for studies with animal models (*n* = 99) or that were irrelevant (*n* = 963), 12 studies met the inclusion criteria (Rotenstreich et al., 2019; Tao et al., 2019; Shi et al., 2020; Uchida et al., 2020; Zhao et al., 2020; Donix et al., 2021; Galvin et al., 2021; Sergott et al., 2021; López-Cuenca et al., 2022; Mathew et al., 2022; Ueda et al., 2022; Wang et al., 2022), among which three studies were excluded after reading the full text for the following reasons: the coefficient of two studies (Ueda et al., 2022; Wang et al., 2022) was calculated in a linear regression model and the number of AD participants in one study was missing (Galvin et al., 2021).

Table 1 summarizes the nine studies in this meta-analysis. All of the nine studies were high-quality studies (Table 2). One cohort study (Sergott et al., 2021) observed macular grid RNFL thickness and hippocampal volume change from baseline to 78 weeks in one randomized controlled trial of Verubecestat. We extracted the Pearson correlation coefficient *r* of the baseline when all patients were recruited without any intervention.

**TABLE 1:** Demographic figures of included studies.

References	Subject	N (M/F)	Age	Cognition assessment	Retina layer	Correlation coefficient *r*	Side(s)	OCT measurements	Structural MRI
Shi et al., 2020	NC	80 (39/41)	68 ± 5.3	MMSE 28 ± 0.2	pRNFL	0.21	double	HD-OCT	T1-weighted 3T
Sergott et al., 2021	AD	1,785 (801/985)	71.7 ± 7.5	MMSE (15–26)	mRNFL	0.102 (*n* = 960)	right	SD-OCT	T1-weighted 1.5 T or 3 T
						0.116 (*n* = 951)	left		
					Retina	0.145 (*n* = 1,111)	right		
						0.117 (*n* = 1,060)	left		
Rotenstreich et al., 2019	AD	64	N/A	Executive function; Episodic memory tests	GCL	0.313	right	SD-OCT	3T
					RPE	0.272			
	NC	23			RPE	−0.47	left		
Uchida et al., 2020	NC	31 (11/20)	65.1 ± 7.6	MoCA 27 (25–28.5)	RPE	−0.393	double	SD-OCT	T1-weighted 3T
Donix et al., 2021	NC	12 (7/5)	65.1 ± 9.0	MMSE 29.3 ± 0.7	pRNFL	0.803	left	Spectralis OCT	T1-weighted 3T
						0.818	right		
Tao et al., 2019	AD MCI**** NC	42 (29/44)**** 48**** (20/31)**** 45**** (24/43)	71.40 ± 7.82 71.67 ± 8.04**** 68.91 ± 5.88	MMSE 19.67 ± 4.58**** 28.33 ± 1.55**** 28.67 ± 1.00	pRNFL	0.302	left		
								OCT	T1-weighted 3T
Zhao et al., 2020	AD	17 (9/8)	70.24 ± 7.53	MMSE 21.18 ± 3.09	mRNFL	0.529	double		
	MCI	23 (12/11)	68.43 ± 5.70	26.91 ± 1.47				Stratus OCT	T1-weighted 3T
	NC	19 (8/11)	66.63 ± 6.17	28.79 ± 1.03					
López-Cuenca et al., 2022	NC	30 (12/18)	60.0 (54.0–64.5)	MMSE 29.0 (29.0–29.0)	IPL	−0.542	right	Spectralis OCT	T1-weighted 1.5T
						−0.595	left		
					INL	−0.422	left		
Mathew et al., 2022	AD	4 (3/1)	68.6 ± 12.0	MoCA 15.5 ± 7.0	pRNFL	0.320	right		T1-weighted 3T
	MCI	17 (10/7)	73.8 ± 7.5	20.6 ± 4.0				HD-OCT	
	SCD	26 (11/15)	71.0 ± 5.6	25.4 ± 3.8		0.306	left		
	NC	28 (6/22)	70.5 ± 5.8	26.4 ± 2.1					

Subjects: AD, Alzheimer’s disease; MCI, mild cognitive impairment; SCD, subjective cognitive decline; NC, Normal control; MMSE, Mini-Mental State Examination; MoCA, Montreal Cognitive Assessment; M = Male; F, Female; pRNFL, Peripapillary retinal nerve fiber layer; mRNFL, Macula retinal nerve fiber layer; GCL, Ganglion cell; IPL, Inner plexiform layer; INL, Inner nuclear layer; OCT, Optical coherence tomography; SD, Spectral-domain; HD, Heidelberg Spectralis; MRI, Magnetic resonance imaging; T, Tesla; N/A, not applicable.

**TABLE 2:** The Newcastle-Ottawa Quality Assessment Scale.

References	Selection				Comparability	Exposure			Scores
	Adequate definition of cases	Representativeness of the cases	Selection of controls	Definition of controls	Control for important factor*	Ascertainment of exposure	Same method of ascertainment of exposure	Non-response rate	
Shi et al., 2020	*	*	*	–	**	*	*	–	7
Sergott et al., 2021	*	*	*	*	**	*	*	–	8
Rotenstreich et al., 2019	*	*	*	*	**	*	*	–	8
Uchida et al., 2020	*	*	*	*	*	*	*	–	7
Donix et al., 2021	*	*	*	*	*	*	*	–	7
Tao et al., 2019	*	*	*	*	*	*	*	–	7
Zhao et al., 2020	*	*	*	*	*	*	*	–	7
López-Cuenca et al., 2022	*	*	*	*	**	*	*	–	8
Mathew et al., 2022	*	*	*	*	*	*	*	–	7

* A maximum of two stars can be allotted in this category, one for age, the other for other controlled factors.

In total, the nine selected studies yielded 18 effect sizes and 4,802 participants. These participants were either MCI/AD patients or normal controls. In one study (Mathew et al., 2022), participants of subjective cognitive decline (SCD) were included. Since SCD is a subjective clinical symptom but not a clinical diagnosis, we chose to combine the data with the normal control group. Six of the nine studies investigated the RNFL (four pRNFL and two mRNFL), two studies reported RPE thickness changes, and one study reported IPL and INL thickness changes.

### Association between retinal thickness and hippocampal volume

**FIGURE 2:**
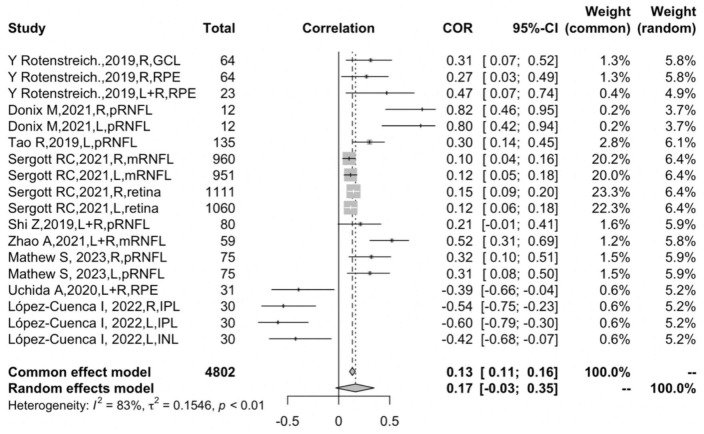
Forest plot for the overall result.

**FIGURE 3:**
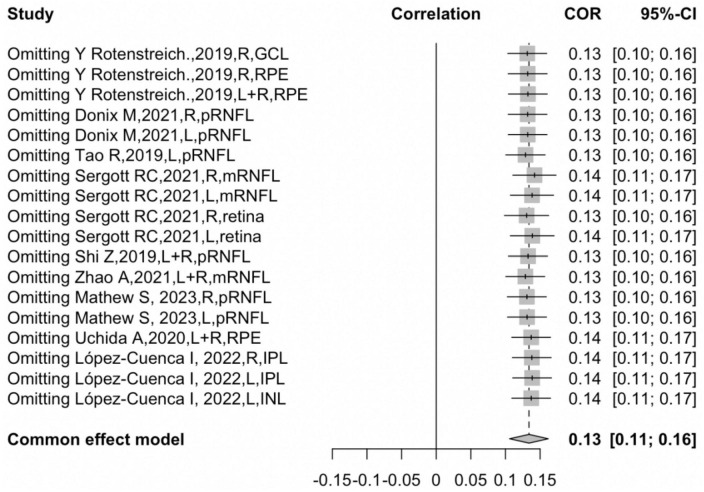
Sensitivity analysis.

Figure 2 shows the forest plot for an insignificant correlation between retinal thickness and hippocampal volume loss (*r* = 0.1651, 95% CI: −0.0288–0.3470, *p* = 0.09). Egger’s regression test revealed a rather symmetrical result (*p* = 0.5968), presenting a low risk of reporting bias. Furthermore, no outliers were identified in an influential analysis that could nullify the correlation between retinal thickness and hippocampal volume in the leave-one-out method (Figure 3). Moreover, high heterogeneity was detected in the overall samples (*I*^2^ = 82.5%, *p* < 0.01).

### Moderator effects

In order to investigate the potential sources of heterogeneity, subgroup analysis was conducted with several moderator variables, including participants, hemisphere, and retinal layers.

### The correlation between retinal thickness and hippocampal volume in AD, MCI, and NC subjects

**FIGURE 4:**
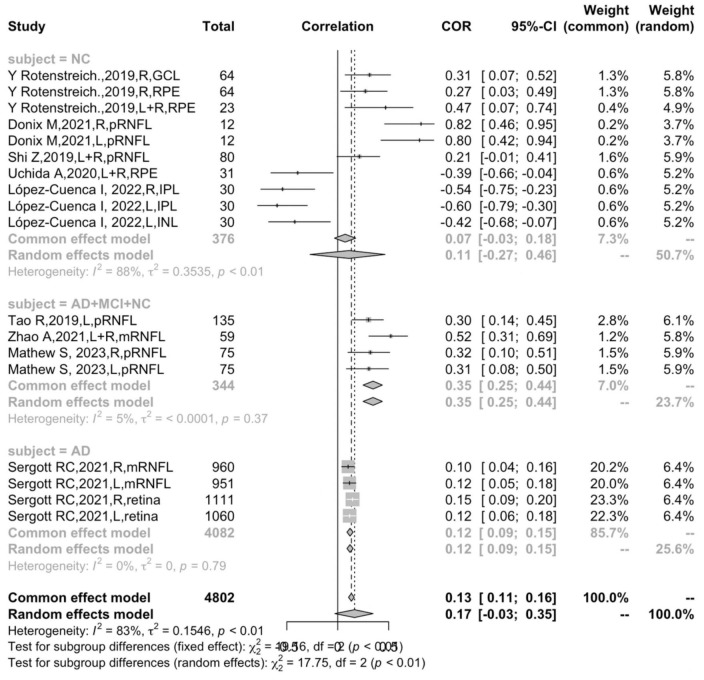
Subgroup analysis of different participants.

In the groups of different participants, the correlation between total retinal thickness and hippocampal volume was significantly positively correlated in AD patients (*r* = 0.1209, 95% CI:0.0905–0.1510, *I*^2^ = 0.0%), and for participants of AD, MCI, and NC in sum, the correlation was significantly positive (*r* = 0.3469, 95% CI: 0.2490–0.4377, *I*^2^ = 5.0%). It was discovered that the difference between the AD and NC groups was significant (*P* < 0.05). For the NC group, however, the correlation was positive but rather unstable (*r* = 0.1114, 95% CI: −0.2718–0.4641, *I*^2^ = 87.8%), revealing a high heterogeneity, suggesting that the participant was not the only moderator that accounted for heterogeneity (Figure 4).

### The correlation between different retinal layers and hippocampal volume

**FIGURE 5:**
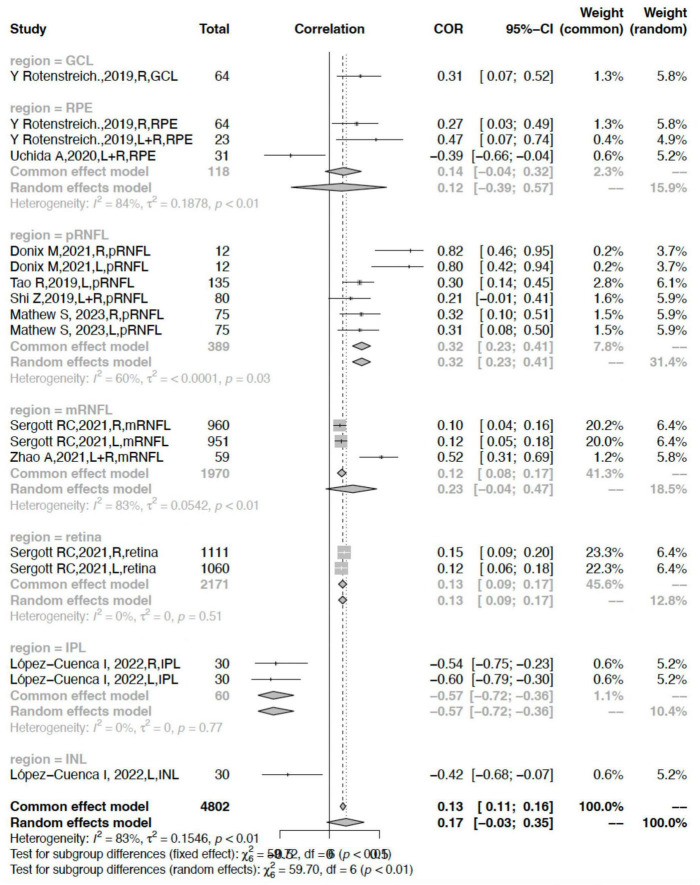
Subgroup analysis of different retinal layers.

In all, RNFL thickness was reported in six studies (Tao et al., 2019; Shi et al., 2020; Zhao et al., 2020; Donix et al., 2021; Sergott et al., 2021; Mathew et al., 2022), RPE change was reported in two studies (Rotenstreich et al., 2019; Uchida et al., 2020), and GCL and IPL/INL each in one study separately (Rotenstreich et al., 2019; López-Cuenca et al., 2022). In the subgroup of different retinal layers, the pRNFL showed a stable moderate positive correlation (*r* = 0.3242, 95% CI: 0.2303–0.4120, *I*^2^ = 60.1%) (Figure 5).

In comparison, the correlation between the mRNFL or RPE and hippocampal volume was rather weak (*r* = 0.1222, 95% CI: 0.0784–0.1655, *I*^2^ = 83.4%; *r* = 0.1421, 95% 0.0447–0.3192, *I*^2^ = 84.1%) showing heavy heterogeneity. Other parameters including the retina and IPL had a weak correlation and low heterogeneity (retina: *r* = 0.1314, 95% CI: 0.0898–0.1725, *I*^2^ = 0.0%; IPL: *r* = −0.5691, 95% CI: −0.7225- −0.3622, *I*^2^ = 0.0%) (Figure 5).

**FIGURE 6:**
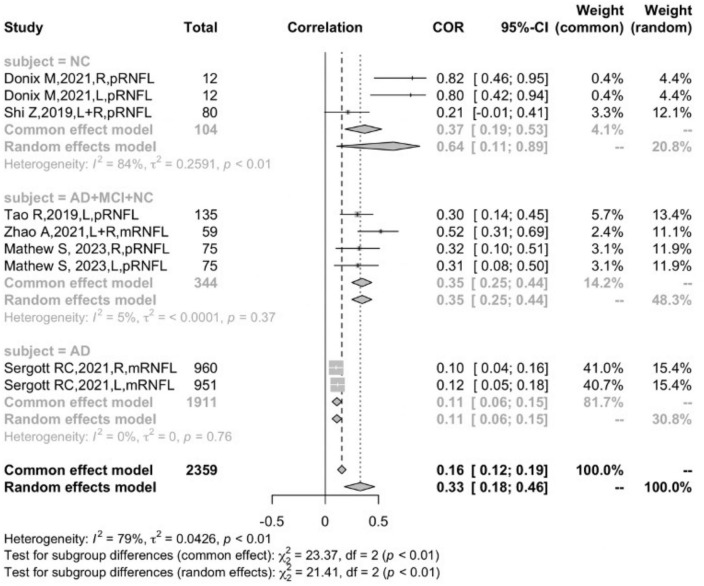
Subgroup analysis of the RNFL.

In the six studies that reported RNFL thickness, the correlation between the RNFL and hippocampal volume in the NC group (*r* = 0.6407, 95% CI: 0.1084–0.8875, *I*^2^ = 83.6%) was significantly stronger than that of AD (*r* = 0.1090, 95% CI: 0.0644−0.1531, *I*^2^ = 0%) (*P* < 0.05) (Figure 6).

### The correlation between retinal thickness and hippocampal volume in different hemispheres

**FIGURE 7:**
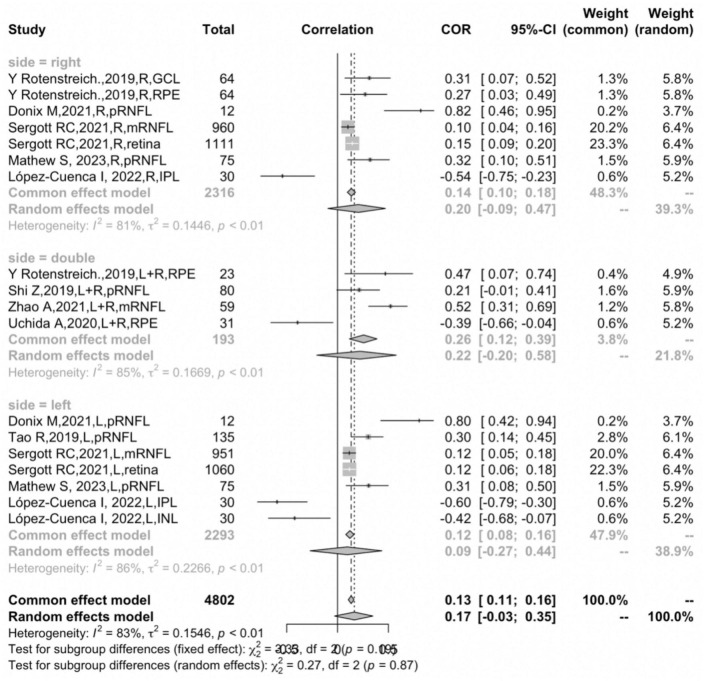
Subgroup analysis of different hemispheres.

In the subgroup analysis that measured hemisphere sides, four studies (Rotenstreich et al., 2019; Shi et al., 2020; Uchida et al., 2020; Zhao et al., 2020) reported the correlation coefficient bilaterally, and six studies (Rotenstreich et al., 2019; Tao et al., 2019; Donix et al., 2021; Sergott et al., 2021; López-Cuenca et al., 2022; Mathew et al., 2022) reported the correlation coefficient of the left and right side separately. Further subgroup analysis suggested that measuring sides was not the main factor leading to heterogeneity (*P* = 0.1889) (Figure 7). Due to the absence of the age variable in one study (Rotenstreich et al., 2019), and the effect size did not categorize by gender, subgroup analysis could not be conducted using these two variables.

### Meta-regression

The moderator effect analysis suggested that any variables of the subject, retinal layer, or hemisphere could not be the independent source that eliminates all heterogeneity. Therefore, we implemented meta-regression analysis to accommodate for the unaccounted heterogeneity. The meta-regression analysis revealed that in the overall studies, the variable of the retinal layer alone accounted for 65.55% of all heterogeneity. Furthermore, the two variables of the subject together with the retinal layers accounted for 100.00% of all heterogeneity.

## Discussion

This is the first study so far to conduct a meta-analysis of the correlation between retinal thickness and hippocampal volume. Through the combined statistics of nine studies on the OCT detection of retinal thickness and volumetric MRI of hippocampal atrophy, the correlation between retinal changes and hippocampal atrophy in AD patients and cognitively normal participants was explored. The main finding of this study is that there is a significant positive correlation between retinal thickness and hippocampal volume in AD participants (*r* = 0.1209, 95% CI:0.0905–0.1510, *I*^2^ = 0.0%). In the participants of AD, MCI, and NC in sum, a positive correlation was detected (*r* = 0.3469, 95% CI: 0.2490–0.4377, *I*^2^ = 5.0%). It is worth noting that the retinal layer of focus in three participant groups was the RNFL, suggesting that the correlation between RNFL thickness and hippocampal atrophy was consistent from NC to AD.

A high degree of heterogeneity was detected in the statistical analysis of this study, suggesting that the overall pooled results may not be present in all cases. Given that the heterogeneity is most likely due to the clinical heterogeneity of the subjects in the original study, this suggests the diversity of the subjects can greatly affect the accuracy of the statistical results. It was suggested that different retinal layers contribute most to the heterogeneity; second to this, participants were the other moderator factor that accounted for the residual heterogeneity. The study by Sergott et al. (2021), contributed to more than half of the total sample size and greatly affected the results of the overall or subgroup analysis, even though Egger’s test did not reveal a publication bias. Methodologically, the precision of the effect size is inversely related to sample size (Sedgwick and Marston, 2015). Correspondingly, sample size is also one of the independent factors that can affect heterogeneity.

The subgroup analysis and meta-regression demonstrated that the participants were an independent factor affecting the correlation between retinal thickness and hippocampal volume. Interestingly, the correlation of all participants, including AD, MCI, and NC (*r* = 0.3469, 95% CI: 0.2490–0.4377, *I*^2^ = 5.0%), was significantly stronger than that of the AD group (*r* = 0.1209, 95% CI:0.0905–0.1510, *I*^2^ = 0.0%), and the difference between the two groups was statistically significant (*P* < 0.0001) (Figure 4). One possible explanation for this might be the fact that retinal attenuation appears to be different between normal aging and AD pathology. Previous meta-analyses have suggested retinal thickness was significantly decreased in AD patients, but the difference was rather small compared with the normal controls (den Haan et al., 2017; Chan et al., 2019). Coincidentally, den Haan et al. (2019) indicated a statistically significant retinal thinning in well-characterized AD but failed to discriminate from normal aging in a cross-sectional study. Several studies also indicated that the retina–brain association became significant when adjusted for age, gender, education, etc., (Liu et al., 2016; Casaletto et al., 2017). This evidence suggests that retinal thickness might be informative in non-demented populations or AD patients at an early stage, but not strong enough to be an independent biomarker for AD. We speculate that the atrophy in the retina might not be unanimous in all ten layers since the IPL and INL thickness were negatively correlated to hippocampal volume.

It was certain that there was a positive association between RNFL thickness and hippocampal volume loss in both NC and the AD continuum. However, our subgroup suggested that the correlation of RNFL thickness and hippocampal atrophy in the NC group was much higher than that of the AD group (NC: *r* = 0.6407, 95% CI:0.1084–0.8875; AD: *r* = 0.1090, 95% CI:0.0644–0.1531) (Figure 6). In previous studies (Kesler et al., 2011; Shen et al., 2014; Cheung et al., 2015; Gao et al., 2015), a large amount of data have shown that the RNFL has a relatively obvious atrophy phenomenon in AD patients. Chan et al. (2019) conducted a meta-analysis of 30 studies and showed that the spectral-domain OCT (SD-OCT) measurements of the inner retina of AD patients (including macular ganglion cell-inner plexiform layer (GC-IPL) thickness, macular ganglion cell complex (GCC) thickness, and peripapillary RNFL thickness) were significantly thinner than those of controls. At the same time, retinal macular thickness and macular volume were also significantly decreased in AD patients compared with controls. In two (Ko et al., 2018; Mutlu et al., 2018) large-scale cohort studies, the results suggested that RNFL thinning predicted the decline in cognitive level and increased risk of AD in patients, and this conclusion was still statistically significant after adjusting for cardiovascular risk factors. den Haan et al. (2017), Chan et al. (2019) conducted a systematic review and meta-analysis of 25 studies measuring retinal thickness and found that compared with NC, the average peripapillary RNFL and macular thickness of AD patients were reduced, but the study also suggested that the small between-group difference in RNFL thinning might limit its potential as a biomarker. Still, other studies (Lehmann et al., 2013; Mutlu et al., 2018) have shown that visual ratings are somewhat correlated with structures in the medial temporal lobe, but not the hippocampus. Casaletto et al. (2017) found that in an elderly population with normal cognition, there was a linear correlation between RNFL and entorhinal cortex volume, which was statistically significant; however, there was no significant correlation between the hippocampus and any retinal structure, while the para-hippocampus structure had a significant correlation with RNFL thinning. According to the differences between groups in this meta-analysis, the correlation between RNFL thickness and hippocampal volume in the NC group (*r* = 0.6407, 95% CI: 0.1084–0.8875, *I*^2^ = 83.6%) was significantly stronger than that of AD (*r* = 0.1090, 95% CI: 0.0644–0.1531, *I*^2^ = 0%), suggesting that AD pathology might alter the changes of the retinal structure and it no longer has the same atrophy progression as the NC group. The average age of participants included in the NC group was above 65 years old, thus we consider that the specific atrophy of the RNFL might occur through aging. Synchronously, considering that the correlation belongs to the consistency of the distribution between the two variables, therefore, the pattern of retinal volume changes in AD patients may be different from the process of hippocampal atrophy, and this view needs to be further proved at the pathological level.

This study also found that RPE volume loss had a positive correlation with hippocampal atrophy (*r* = 0.1421, 95% CI:−0.0447–0.3192], *I*^2^ = 84.1%) (Figure 5). In previous studies, no significant categorical changes in outer retinal thickness were found in subjects with neurodegenerative diseases, but there was a correlation between outer retinal loss and neurocognitive decline (den Haan et al., 2018a; Uchida et al., 2020). The RPE is the epithelium of the outer layer of the retina, and since glaucoma is characterized by thinning of the retinal nerve fiber layer or other inner retinal diseases, it is theoretically less affected by the presence of glaucoma (Chen et al., 2017) and more closely associated with neurodegeneration. Previous studies generally believed that the inner layer of the retina has a greater correlation with cognitive decline in neurodegenerative diseases and hippocampal atrophy (Gordon-Lipkin et al., 2007), but the inner layer of the retina may often be affected by glaucoma and other diseases. In terms of the RPE being the outermost layer of the retina, the RPE presents a weak positive correlation.

The possible pathological mechanisms underlying the association between RPE volume and hippocampal atrophy are unclear. Löffler et al. (1995) discovered the Aβ immunoreactivity in the RPE of normal elderly people. Toxic Aβ oligomers were found to be aggregates in drusen of macular RPE (Johnson et al., 2002; Luibl et al., 2006). Drusen-like Aβ deposits were also found in the RPE of 5xFAD mice (Park et al., 2014). Other possible mechanisms might be related to the high abundance of long-chain polyunsaturated fatty acids (PUFA), especially docosahexaenoic acid (DHA, 22:6n-3), which accounts for the outer segments of photoreceptors and the main structure of lipids in the cortex (SanGiovanni and Chew, 2005). DHA, as an important component of biofilms in neuronal tissues including the retina, plays a key role in maintaining the flexibility of the bilayer as well as the renewal of the outer segment membranes of photoreceptors (SanGiovanni and Chew, 2005). Evidence from a recent study showed that the total amount of DHA and eicosatetraenoic acid in erythrocytes was positively correlated with whole brain volume in adult subjects (Pottala et al., 2014). Some scholars believe that the chronic reduction of DHA in the retina is related to the atrophy of the RPE. This may be due to age-related decreased DHA uptake and transport to photoreceptors through the choroidal capillary-RPE. Retinal lipid synthesis may also be affected by desaturase activation or antioxidant systems. But, under normal conditions, DHA can also be efficiently recycled in the retina and the RPE (SanGiovanni and Chew, 2005).

There were several limitations of our meta-analysis: first, while four of the nine studies investigated AD patients, three of them pooled the AD data with MCI and NC groups, and we did not have access to the data of AD patients independently. The independent retinal thickness of AD was mainly detected in one single study. Second, 90% of AD patients in our research were clinically diagnosed. A lack of Aβ or tau biomarkers might lead to case misclassification. Furthermore, heterogeneity in sample size preserves obvious differences in the statistical analytical results, which might affect precision. Thus, larger sample sizes studies with meticulously designed methods are necessary for validation.

## Conclusion

This meta-analysis revealed a positive correlation between retinal thickness and hippocampal atrophy. The correlation appears to be more predominant in the RNFL than in other layers, especially the RNFL in NC groups rather than AD groups, suggesting that retinal thinning appears along with aging and probably initiates in the early stages of the continuum of AD.

## Data availability statement

The raw data supporting the conclusions of this article will be made available by the authors, without undue reservation.

## Author contributions

QM and MS: concept and design. SC and DZ: literature search. HZ, MS, and TC: statistical analysis. SC and KX: manuscript writing. All authors critical revision of the manuscript for intellectual content.

## Footnotes

1
https://rstudio.com/

## References

[B1] Albert M. S. DeKosky S. T. Dickson D. Dubois B. Feldman H. H. Fox N. C. (2011). The diagnosis of mild cognitive impairment due to Alzheimer’s disease: Recommendations from the National Institute on Aging-Alzheimer’s Association workgroups on diagnostic guidelines for Alzheimer’s disease. *Alzheimers Dement.* 7 270–279. 10.1016/j.jalz.2011.03.008 21514249 PMC3312027

[B2] Alzheimer’s Association (2020). 2020 Alzheimer’s disease facts and figures. *Alzheimers Dement*. 10.1002/alz.12068 [Epub ahead of print]. 32157811

[B3] Berk C. Paul G. Sabbagh M. (2014). Investigational drugs in Alzheimer’s disease: Current progress. *Expert Opin. Investig. Drugs* 23 837–846. 10.1517/13543784.2014.905542 24702504

[B4] Berry K. J. Mielke P. W. Jr. (2000). A Monte Carlo investigation of the Fisher Z transformation for normal and nonnormal distributions. *Psychol. Rep.* 87 1101–1114. 10.2466/pr0.2000.87.3f.1101 11272750

[B5] Casaletto K. B. Ward M. E. Baker N. S. Bettcher B. M. Gelfand J. M. Li Y. (2017). Retinal thinning is uniquely associated with medial temporal lobe atrophy in neurologically normal older adults. *Neurobiol. Aging* 51 141–147. 10.1016/j.neurobiolaging.2016.12.011 28068565 PMC5554591

[B6] Chan V. T. T. Sun Z. Tang S. Chen L. J. Wong A. Tham C. C. (2019). Spectral-domain OCT measurements in Alzheimer’s disease: A systematic review and meta-analysis. *Ophthalmology* 126 497–510. 10.1016/j.ophtha.2018.08.009 30114417 PMC6424641

[B7] Chen Q. Huang S. Ma Q. Lin H. Pan M. Liu X. (2017). Ultra-high resolution profiles of macular intra-retinal layer thicknesses and associations with visual field defects in primary open angle glaucoma. *Sci. Rep.* 7:41100. 10.1038/srep41100 28169283 PMC5294583

[B8] Cheung C. Y. Ikram M. K. Chen C. Wong T. Y. (2017). Imaging retina to study dementia and stroke. *Prog. Retin. Eye Res.* 57 89–107. 10.1016/j.preteyeres.2017.01.001 28057562

[B9] Cheung C. Y. Ong Y. T. Hilal S. Ikram M. K. Low S. Ong Y. L. (2015). Retinal ganglion cell analysis using high-definition optical coherence tomography in patients with mild cognitive impairment and Alzheimer’s disease. *J. Alzheimers Dis.* 45 45–56. 10.3233/JAD-141659 25428254

[B10] Cunha J. P. Proença R. Dias-Santos A. Almeida R. Águas H. Alves M. (2017). OCT in Alzheimer’s disease: Thinning of the RNFL and superior hemiretina. *Graefes Arch. Clin. Exp. Ophthalmol.* 255 1827–1835. 10.1007/s00417-017-3715-9 28643042

[B11] Cunha L. P. Almeida A. L. Costa-Cunha L. V. Costa C. F. Monteiro M. L. (2016). The role of optical coherence tomography in Alzheimer’s disease. *Int. J. Retina Vitreous* 2:24. 10.1186/s40942-016-0049-4 27847642 PMC5088456

[B12] den Haan J. Csinscik L. Parker T. Paterson R. W. Slattery C. F. Foulkes A. (2019). Retinal thickness as potential biomarker in posterior cortical atrophy and typical Alzheimer’s disease. *Alzheimers Res. Ther.* 11:62. 10.1186/s13195-019-0516-x 31319885 PMC6639972

[B13] den Haan J. Janssen S. F. van de Kreeke J. A. Scheltens P. Verbraak F. D. Bouwman F. H. (2018a). Retinal thickness correlates with parietal cortical atrophy in early-onset Alzheimer’s disease and controls. *Alzheimers Dement.* 10 49–55. 10.1016/j.dadm.2017.10.005 29201990 PMC5699891

[B14] den Haan J. Morrema T. H. J. Verbraak F. D. de Boer J. F. Scheltens P. Rozemuller A. J. (2018b). Amyloid-beta and phosphorylated tau in post-mortem Alzheimer’s disease retinas. *Acta Neuropathol. Commun.* 6:147. 10.1186/s40478-018-0650-x 30593285 PMC6309096

[B15] den Haan J. Verbraak F. D. Visser P. J. Bouwman F. H. (2017). Retinal thickness in Alzheimer’s disease: A systematic review and meta-analysis. *Alzheimers Dement.* 6 162–170. 10.1016/j.dadm.2016.12.014 28275698 PMC5328759

[B16] Donix M. Wittig D. Hermann W. Haussmann R. Dittmer M. Bienert F. (2021). Relation of retinal and hippocampal thickness in patients with amnestic mild cognitive impairment and healthy controls. *Brain Behav* 11:e02035. 10.1002/brb3.2035 33448670 PMC8119792

[B17] Dubois B. Feldman H. H. Jacova C. Dekosky S. T. Barberger-Gateau P. Cummings J. (2007). Research criteria for the diagnosis of Alzheimer’s disease: Revising the NINCDS-ADRDA criteria. *Lancet Neurol.* 6 734–746. 10.1016/S1474-4422(07)70178-3 17616482

[B18] Fox N. C. Scahill R. I. Crum W. R. Rossor M. N. (1999). Correlation between rates of brain atrophy and cognitive decline in AD. *Neurology* 52 1687–1689. 10.1212/WNL.52.8.1687 10331700

[B19] Frisoni G. B. Fox N. C. Jack C. R. Jr. Scheltens P. Thompson P. M. (2010). The clinical use of structural MRI in Alzheimer disease. *Nat. Rev. Neurol.* 6 67–77. 10.1038/nrneurol.2009.215 20139996 PMC2938772

[B20] Galvin J. E. Kleiman M. J. Walker M. (2021). Using optical coherence tomography to screen for cognitive impairment and dementia. *J. Alzheimers Dis.* 84 723–736. 10.3233/JAD-210328 34569948 PMC10731579

[B21] Gao L. Liu Y. Li X. Bai Q. Liu P. (2015). Abnormal retinal nerve fiber layer thickness and macula lutea in patients with mild cognitive impairment and Alzheimer’s disease. *Arch. Gerontol. Geriatr.* 60 162–167. 10.1016/j.archger.2014.10.011 25459918

[B22] Ge Y. J. Xu W. Ou Y. N. Qu Y. Ma Y. H. Huang Y. Y. (2021). Retinal biomarkers in Alzheimer’s disease and mild cognitive impairment: A systematic review and meta-analysis. *Ageing Res. Rev.* 69:101361. 10.1016/j.arr.2021.101361 34000463

[B23] Gordon-Lipkin E. Chodkowski B. Reich D. S. Smith S. A. Pulicken M. Balcer L. J. (2007). Retinal nerve fiber layer is associated with brain atrophy in multiple sclerosis. *Neurology* 69 1603–1609. 10.1212/01.wnl.0000295995.46586.ae 17938370

[B24] Grimaldi A. Pediconi N. Oieni F. Pizzarelli R. Rosito M. Giubettini M. (2019). Neuroinflammatory processes, A1 astrocyte activation and protein aggregation in the retina of Alzheimer’s disease patients, possible biomarkers for early diagnosis. *Front. Neurosci.* 13:925. 10.3389/fnins.2019.00925 31551688 PMC6737046

[B25] Jack C. R. Jr. Bennett D. A. Blennow K. Carrillo M. C. Dunn B. Haeberlein S. B. (2018). Toward a biological definition of Alzheimer’s disease. *Alzheimers Dement.* 14 535–562. 10.1016/j.jalz.2018.02.018 29653606 PMC5958625

[B26] Jack C. R. Jr. Lowe V. J. Weigand S. D. Wiste H. J. Senjem M. L. Knopman D. S. (2009). Serial PIB and MRI in normal, mild cognitive impairment and Alzheimer’s disease: Implications for sequence of pathological events in Alzheimer’s disease. *Brain* 132 1355–1365. 10.1093/brain/awp062 19339253 PMC2677798

[B27] Jindahra P. Hedges T. R. Mendoza-Santiesteban C. E. Plant G. T. (2010). Optical coherence tomography of the retina: Applications in neurology. *Curr. Opin. Neurol.* 23 16–23. 10.1097/WCO.0b013e328334e99b 20009925

[B28] Johnson L. V. Leitner W. P. Rivest A. J. Staples M. K. Radeke M. J. Anderson D. H. (2002). The Alzheimer’s A beta -peptide is deposited at sites of complement activation in pathologic deposits associated with aging and age-related macular degeneration. *Proc. Natl. Acad. Sci. U.S.A.* 99 11830–11835. 10.1073/pnas.192203399 12189211 PMC129354

[B29] Katsimpris A. Karamaounas A. Sideri A. M. Katsimpris J. Georgalas I. Petrou P. (2022). Optical coherence tomography angiography in Alzheimer’s disease: A systematic review and meta-analysis. *Eye* 36 1419–1426. 10.1038/s41433-021-01648-1 34193983 PMC9232568

[B30] Kesler A. Vakhapova V. Korczyn A. D. Naftaliev E. Neudorfer M. (2011). Retinal thickness in patients with mild cognitive impairment and Alzheimer’s disease. *Clin. Neurol. Neurosurg.* 113 523–526. 10.1016/j.clineuro.2011.02.014 21454010

[B31] Ko F. Muthy Z. A. Gallacher J. Sudlow C. Rees G. Yang Q. (2018). Association of retinal nerve fiber layer thinning with current and future cognitive decline: A study using optical coherence tomography. *JAMA Neurol.* 75 1198–1205. 10.1001/jamaneurol.2018.1578 29946685 PMC6233846

[B32] Koronyo Y. Biggs D. Barron E. Boyer D. S. Pearlman J. A. Au W. J. (2017). Retinal amyloid pathology and proof-of-concept imaging trial in Alzheimer’s disease. *JCI Insight* 2:e93621. 10.1172/jci.insight.93621 28814675 PMC5621887

[B33] Larrosa J. M. Garcia-Martin E. Bambo M. P. Pinilla J. Polo V. Otin S. (2014). Potential new diagnostic tool for Alzheimer’s disease using a linear discriminant function for Fourier domain optical coherence tomography. *Invest. Ophthalmol. Vis. Sci.* 55 3043–3051. 10.1167/iovs.13-13629 24736054

[B34] Leger F. Fernagut P. O. Canron M. H. Léoni S. Vital C. Tison F. (2011). Protein aggregation in the aging retina. *J. Neuropathol. Exp. Neurol.* 70 63–68. 10.1097/NEN.0b013e31820376cc 21157377

[B35] Lehmann M. Koedam E. L. Barnes J. Bartlett J. W. Barkhof F. Wattjes M. P. (2013). Visual ratings of atrophy in MCI: Prediction of conversion and relationship with CSF biomarkers. *Neurobiol. Aging* 34 73–82. 10.1016/j.neurobiolaging.2012.03.010 22516280

[B36] Liu S. Ong Y. T. Hilal S. Loke Y. M. Wong T. Y. Chen C. L. (2016). The association between retinal neuronal layer and brain structure is disrupted in patients with cognitive impairment and Alzheimer’s disease. *J. Alzheimers Dis.* 54 585–595. 10.3233/JAD-160067 27567815

[B37] Löffler K. U. Edward D. P. Tso M. O. (1995). Immunoreactivity against tau, amyloid precursor protein, and beta-amyloid in the human retina. *Invest. Ophthalmol. Vis. Sci.* 36 24–31. 7822152

[B38] London A. Benhar I. Schwartz M. (2013). The retina as a window to the brain-from eye research to CNS disorders. *Nat. Rev. Neurol.* 9 44–53. 10.1038/nrneurol.2012.227 23165340

[B39] López-Cuenca I. Marcos-Dolado A. Yus-Fuertes M. Salobrar-García E. Elvira-Hurtado L. Fernández-Albarral J. A. (2022). The relationship between retinal layers and brain areas in asymptomatic first-degree relatives of sporadic forms of Alzheimer’s disease: An exploratory analysis. *Alzheimers Res. Ther.* 14:79. 10.1186/s13195-022-01008-5 35659054 PMC9166601

[B40] Luibl V. Isas J. M. Kayed R. Glabe C. G. Langen R. Chen J. (2006). Drusen deposits associated with aging and age-related macular degeneration contain nonfibrillar amyloid oligomers. *J. Clin. Invest.* 116 378–385. 10.1172/JCI25843 16453022 PMC1359048

[B41] Mathew S. WuDunn D. Mackay D. D. Vosmeier A. Tallman E. F. Deardorff R. (2022). Association of brain volume and retinal thickness in the early stages of Alzheimer’s disease. *J. Alzheimers Dis*. 91, 743–752. 10.3233/JAD-210533 36502316 PMC9990456

[B42] Moher D. Liberati A. Tetzlaff J. Altman D. G. (2010). Preferred reporting items for systematic reviews and meta-analyses: The PRISMA statement. *Int. J. Surg.* 8 336–341. 10.1016/j.ijsu.2010.02.007 20171303

[B43] Morra J. H. Tu Z. Apostolova L. G. Green A. E. Avedissian C. Madsen S. K. (2009). Automated 3D mapping of hippocampal atrophy and its clinical correlates in 400 subjects with Alzheimer’s disease, mild cognitive impairment, and elderly controls. *Hum. Brain Mapp.* 30 2766–2788. 10.1002/hbm.20708 19172649 PMC2733926

[B44] Mutlu U. Colijn J. M. Ikram M. A. Bonnemaijer P. W. M. Licher S. Wolters F. J. (2018). Association of retinal neurodegeneration on optical coherence tomography with dementia: A population-based study. *JAMA Neurol.* 75 1256–1263. 10.1001/jamaneurol.2018.1563 29946702 PMC6233847

[B45] Park S. W. Kim J. H. Mook-Jung I. Kim K. W. Park W. J. Park K. H. (2014). Intracellular amyloid beta alters the tight junction of retinal pigment epithelium in 5XFAD mice. *Neurobiol. Aging* 35 2013–2020. 10.1016/j.neurobiolaging.2014.03.008 24709310

[B46] Pottala J. V. Yaffe K. Robinson J. G. Espeland M. A. Wallace R. Harris W. S. (2014). Higher RBC EPA + DHA corresponds with larger total brain and hippocampal volumes: WHIMS-MRI study. *Neurology* 82 435–442. 10.1212/WNL.0000000000000080 24453077 PMC3917688

[B47] Ridha B. H. Anderson V. M. Barnes J. Boyes R. G. Price S. L. Rossor M. N. (2008). Volumetric MRI and cognitive measures in Alzheimer disease: Comparison of markers of progression. *J. Neurol.* 255 567–574. 10.1007/s00415-008-0750-9 18274807

[B48] Rotenstreich Y. Sharvit-Ginon I. Zloto O. Fabian I. D. Elkader A. Abd Beeri M. (2019). Association of brain structure and cognitive function with structural retinal markers in asymptomatic individuals at high risk for Alzheimer disease. *Investig. Ophthalmol. Vis. Sci.* 60:1878.

[B49] SanGiovanni J. P. Chew E. Y. (2005). The role of omega-3 long-chain polyunsaturated fatty acids in health and disease of the retina. *Prog. Retin. Eye Res.* 24 87–138. 10.1016/j.preteyeres.2004.06.002 15555528

[B50] Sedgwick P. Marston L. (2015). How to read a funnel plot in a meta-analysis. *BMJ* 351 h4718. 10.1136/bmj.h4718 26377337

[B51] Sergott R. C. Raji A. Kost J. Sur C. Jackson S. Locco A. (2021). Retinal optical coherence tomography metrics are unchanged in verubecestat Alzheimer’s disease clinical trial but correlate with baseline regional brain atrophy. *J. Alzheimers Dis.* 79 275–287. 10.3233/JAD-200735 33252075

[B52] Shen Y. Liu L. Cheng Y. Feng W. Shi Z. Zhu Y. (2014). Retinal nerve fiber layer thickness is associated with episodic memory deficit in mild cognitive impairment patients. *Curr. Alzheimer Res.* 11 259–266. 10.2174/1567205011666140131114418 24484274

[B53] Shi Z. Cao X. Hu J. Jiang L. Mei X. Zheng H. (2020). Retinal nerve fiber layer thickness is associated with hippocampus and lingual gyrus volumes in nondemented older adults. *Prog. Neuropsychopharmacol. Biol. Psychiatry* 99:109824. 10.1016/j.pnpbp.2019.109824 31765713

[B54] Sluimer J. D. Bouwman F. H. Vrenken H. Blankenstein M. A. Barkhof F. van der Flier W. M. (2010). Whole-brain atrophy rate and CSF biomarker levels in MCI and AD: A longitudinal study. *Neurobiol. Aging* 31 758–764. 10.1016/j.neurobiolaging.2008.06.016 18692273

[B55] Tábuas-Pereira M. T. Baldeiras I. Duro D. Santiago B. Ribeiro M. H. Leitão M. J. (2016). Prognosis of early-onset vs. late-onset mild cognitive impairment: Comparison of conversion rates and its predictors. *Geriatrics* 1:11. 10.3390/geriatrics1020011 31022805 PMC6371125

[B56] Tao R. Lu Z. Ding D. Fu S. Hong Z. Liang X. (2019). Perifovea retinal thickness as an ophthalmic biomarker for mild cognitive impairment and early Alzheimer’s disease. *Alzheimers Dement.* 11 405–414. 10.1016/j.dadm.2019.04.003 31206006 PMC6558027

[B57] Thompson P. M. Hayashi K. M. De Zubicaray G. I. Janke A. L. Rose S. E. Semple J. (2004). Mapping hippocampal and ventricular change in Alzheimer disease. *Neuroimage* 22 1754–1766. 10.1016/j.neuroimage.2004.03.040 15275931

[B58] Uchida A. Pillai J. A. Bermel R. Jones S. E. Fernandez H. Leverenz J. B. (2020). Correlation between brain volume and retinal photoreceptor outer segment volume in normal aging and neurodegenerative diseases. *PLoS One* 15:e0237078. 10.1371/journal.pone.0237078 32881874 PMC7470418

[B59] Ueda E. Hirabayashi N. Ohara T. Hata J. Honda T. Fujiwara K. (2022). Association of inner retinal thickness with prevalent dementia and brain atrophy in a general older population: The Hisayama study. *Ophthalmol. Sci.* 2:100157. 10.1016/j.xops.2022.100157 36249677 PMC9559916

[B60] Wang R. Kwapong W. R. Tao W. Cao L. Ye C. Liu J. (2022). Association of retinal thickness and microvasculature with cognitive performance and brain volumes in elderly adults. *Front. Aging Neurosci.* 14:1010548. 10.3389/fnagi.2022.1010548 36466601 PMC9709407

[B61] Wells G. A. Shea B. O’Connell D. Peterson J. Welch V. Losos M. (2000). *The Newcastle-Ottawa Scale (NOS) for assessing the quality of nonrandomised studies in meta-analyses.* Oxford.

[B62] Zhao A. Fang F. Li B. Chen Y. Qiu Y. Wu Y. (2020). Visual abnormalities associate with hippocampus in mild cognitive impairment and early Alzheimer’s disease. *Front. Aging Neurosci.* 12:597491. 10.3389/fnagi.2020.597491 33551787 PMC7862343

