# Supplementary material for: The association between retina thinning and hippocampal atrophy in Alzheimer’s disease and mild cognitive impairment: a meta-analysis and systematic review
Source: Front Aging Neurosci. 2023 Aug 23;15:1232941. doi: 10.3389/fnagi.2023.1232941 (PMC10481874; doi:10.3389/fnagi.2023.1232941)
Supplement: Supplementary file 1 [file Table_1.DOCX]

PUBMED

#1"tomography, optical coherence"[MeSH Terms] OR ("tomography"[All Fields] AND "optical"[All Fields] AND "coherence"[All Fields]) OR "optical coherence tomography"[All Fields] OR ("optical"[All Fields] AND "coherence"[All Fields] AND "tomography"[All Fields])

#2 "alzheimer disease"[MeSH Terms] OR ("alzheimer"[All Fields] AND "disease"[All Fields]) OR "alzheimer disease"[All Fields] OR "alzheimer's"[All Fields]

#3 "dementia"[MeSH Terms] OR "dementia"[All Fields]

#4 ("mild"[All Fields] AND "cognitive"[All Fields] AND "impairment"[All Fields]) OR "mild cognitive impairment"[All Fields] OR "MCI"[All Fields]

#5 #2 OR #3 OR #4

#6 #1 AND #5

537 citations

WOS

#1 TS=(Tomography, Optical Coherence OR Coherence Tomography, Optical OR OCT Tomography OR Tomography, OCT OR Optical Coherence Tomography OR OCT)

#2 TS=(Alzheimer Disease OR dementia OR mild cognitive impairment OR MCI)

#3 #1 AND #2

724 citations

EMBASE

Title, Abstract or Author keywords :((Tomography, Optical Coherence OR Coherence Tomography, Optical OR OCT Tomography OR Tomography, OCT OR Optical Coherence Tomography OR OCT) AND (Alzheimer Disease OR dementia OR mild cognitive impairment OR MCI))

587 citations
