# Supplementary material for: The association between retina thinning and hippocampal atrophy in Alzheimer’s disease and mild cognitive impairment: a meta-analysis and systematic review
Source: Front Aging Neurosci. 2023 Aug 23;15:1232941. doi: 10.3389/fnagi.2023.1232941 (PMC10481874; doi:10.3389/fnagi.2023.1232941)
Supplement: Supplementary file 3 [file Table_3.DOCX]

metareg(m1, cor2$region)

Mixed-Effects Model (k = 18; tau^2 estimator: REML)

tau^2 (estimated amount of residual heterogeneity): 0.0532 (SE = 0.0308)

tau (square root of estimated tau^2 value): 0.2308

I^2 (residual heterogeneity / unaccounted variability): 92.33%

H^2 (unaccounted variability / sampling variability): 13.04

R^2 (amount of heterogeneity accounted for): 65.55%

Test for Residual Heterogeneity:

QE(df = 11) = 37.6355, p-val < .0001

Test of Moderators (coefficients 2:7):

QM(df = 6) = 24.7543, p-val = 0.0004

Model Results:

estimate se zval pval ci.lb ci.ub

intrcpt 0.3239 0.2639 1.2273 0.2197 -0.1934 0.8411

cor2$regionINL -0.7740 0.3999 -1.9354 0.0529 -1.5578 0.0098 .

cor2$regionIPL -0.9700 0.3388 -2.8632 0.0042 -1.6341 -0.3060 **

cor2$regionmRNFL -0.0850 0.2988 -0.2846 0.7760 -0.6707 0.5006

cor2$regionpRNFL 0.1085 0.2885 0.3760 0.7069 -0.4570 0.6740

cor2$regionretina -0.1921 0.3110 -0.6176 0.5368 -0.8016 0.4175

cor2$regionRPE -0.2026 0.3130 -0.6473 0.5174 -0.8161 0.4109

---

Signif. codes: 0 ‘***’ 0.001 ‘**’ 0.01 ‘*’ 0.05 ‘.’ 0.1 ‘ ’ 1

> metareg(m1, cor2$subject)

Mixed-Effects Model (k = 18; tau^2 estimator: REML)

tau^2 (estimated amount of residual heterogeneity): 0.1606 (SE = 0.0676)

tau (square root of estimated tau^2 value): 0.4007

I^2 (residual heterogeneity / unaccounted variability): 97.46%

H^2 (unaccounted variability / sampling variability): 39.37

R^2 (amount of heterogeneity accounted for): 0.00%

Test for Residual Heterogeneity:

QE(df = 15) = 78.2000, p-val < .0001

Test of Moderators (coefficients 2:3):

QM(df = 2) = 1.4156, p-val = 0.4927

Model Results:

estimate se zval pval ci.lb ci.ub

intrcpt 0.1206 0.2010 0.6002 0.5484 -0.2733 0.5145

cor2$subjectAD+MCI+NC 0.2619 0.2895 0.9046 0.3657 -0.3056 0.8294

cor2$subjectNC -0.0306 0.2462 -0.1243 0.9011 -0.5131 0.4519

---

Signif. codes: 0 ‘***’ 0.001 ‘**’ 0.01 ‘*’ 0.05 ‘.’ 0.1 ‘ ’ 1

> metareg(m1, cor2$side)

Mixed-Effects Model (k = 18; tau^2 estimator: REML)

tau^2 (estimated amount of residual heterogeneity): 0.1817 (SE = 0.0751)

tau (square root of estimated tau^2 value): 0.4262

I^2 (residual heterogeneity / unaccounted variability): 97.19%

H^2 (unaccounted variability / sampling variability): 35.56

R^2 (amount of heterogeneity accounted for): 0.00%

Test for Residual Heterogeneity:

QE(df = 15) = 94.0239, p-val < .0001

Test of Moderators (coefficients 2:3):

QM(df = 2) = 0.3239, p-val = 0.8505

Model Results:

estimate se zval pval ci.lb ci.ub

intrcpt 0.2254 0.2290 0.9843 0.3250 -0.2234 0.6743

cor2$sideleft -0.1330 0.2862 -0.4646 0.6422 -0.6940 0.4280

cor2$sideright -0.0130 0.2859 -0.0454 0.9638 -0.5733 0.5473

---

Signif. codes: 0 ‘***’ 0.001 ‘**’ 0.01 ‘*’ 0.05 ‘.’ 0.1 ‘ ’ 1

> metareg(m1, cor2$region + cor2$subject + cor2$side)

Mixed-Effects Model (k = 18; tau^2 estimator: REML)

tau^2 (estimated amount of residual heterogeneity): 0.0000 (SE = 0.0014)

tau (square root of estimated tau^2 value): 0.0012

I^2 (residual heterogeneity / unaccounted variability): 0.03%

H^2 (unaccounted variability / sampling variability): 1.00

R^2 (amount of heterogeneity accounted for): 100.00%

Test for Residual Heterogeneity:

QE(df = 7) = 14.0237, p-val = 0.0508

Test of Moderators (coefficients 2:11):

QM(df = 10) = 83.3276, p-val < .0001

Model Results:

estimate se zval pval ci.lb ci.ub

intrcpt -1.6759 0.4898 -3.4218 0.0006 -2.6359 -0.7160 ***

cor2$regionINL -0.7666 0.2331 -3.2883 0.0010 -1.2236 -0.3097 **

cor2$regionIPL -0.9664 0.1875 -5.1549 <.0001 -1.3338 -0.5989 ***

cor2$regionmRNFL 1.2664 0.3572 3.5455 0.0004 0.5663 1.9665 ***

cor2$regionpRNFL 0.4896 0.2069 2.3661 0.0180 0.0840 0.8952 *

cor2$regionretina 1.2890 0.3586 3.5949 0.0003 0.5862 1.9918 ***

cor2$regionRPE 0.0493 0.1739 0.2835 0.7768 -0.2916 0.3902

cor2$subjectAD+MCI+NC 0.9872 0.2064 4.7828 <.0001 0.5827 1.3918 ***

cor2$subjectNC 1.4772 0.3350 4.4093 <.0001 0.8206 2.1338 ***

cor2$sideleft 0.5152 0.1573 3.2750 0.0011 0.2069 0.8236 **

cor2$sideright 0.5226 0.1554 3.3625 0.0008 0.2180 0.8272 ***

---

Signif. codes: 0 ‘***’ 0.001 ‘**’ 0.01 ‘*’ 0.05 ‘.’ 0.1 ‘ ’ 1

> metareg(m1, cor2$region + cor2$subject)

Mixed-Effects Model (k = 18; tau^2 estimator: REML)

tau^2 (estimated amount of residual heterogeneity): 0 (SE = 0.0010)

tau (square root of estimated tau^2 value): 0

I^2 (residual heterogeneity / unaccounted variability): 0.00%

H^2 (unaccounted variability / sampling variability): 1.00

R^2 (amount of heterogeneity accounted for): 100.00%

Test for Residual Heterogeneity:

QE(df = 9) = 25.3471, p-val = 0.0026

Test of Moderators (coefficients 2:9):

QM(df = 8) = 72.0102, p-val < .0001

Model Results:

estimate se zval pval ci.lb ci.ub

intrcpt -0.2156 0.2212 -0.9747 0.3297 -0.6491 0.2179

cor2$regionINL -0.7740 0.2312 -3.3484 0.0008 -1.2270 -0.3209 ***

cor2$regionIPL -0.9700 0.1868 -5.1916 <.0001 -1.3363 -0.6038 ***

cor2$regionmRNFL 0.3250 0.2200 1.4772 0.1396 -0.1062 0.7562

cor2$regionpRNFL 0.0654 0.1641 0.3983 0.6904 -0.2562 0.3869

cor2$regionretina 0.3477 0.2222 1.5646 0.1177 -0.0879 0.7833

cor2$regionRPE -0.1808 0.1599 -1.1308 0.2581 -0.4942 0.1326

cor2$subjectAD+MCI+NC 0.4683 0.1356 3.4541 0.0006 0.2026 0.7340 ***

cor2$subjectNC 0.5395 0.1804 2.9909 0.0028 0.1859 0.8930 **

---

Signif. codes: 0 ‘***’ 0.001 ‘**’ 0.01 ‘*’ 0.05 ‘.’ 0.1 ‘ ’ 1

Warning message:

Fisher scoring algorithm may have gotten stuck at a local maximum.

Setting tau^2 = 0. Check the profile likelihood plot with profile().

> metareg(m1, cor2$region + cor2$side)

Mixed-Effects Model (k = 18; tau^2 estimator: REML)

tau^2 (estimated amount of residual heterogeneity): 0.0854 (SE = 0.0514)

tau (square root of estimated tau^2 value): 0.2922

I^2 (residual heterogeneity / unaccounted variability): 93.28%

H^2 (unaccounted variability / sampling variability): 14.88

R^2 (amount of heterogeneity accounted for): 44.76%

Test for Residual Heterogeneity:

QE(df = 9) = 36.9539, p-val < .0001

Test of Moderators (coefficients 2:9):

QM(df = 8) = 19.0376, p-val = 0.0147

Model Results:

estimate se zval pval ci.lb ci.ub

intrcpt 0.2524 0.3944 0.6399 0.5222 -0.5206 1.0255

cor2$regionINL -0.6957 0.5115 -1.3602 0.1738 -1.6983 0.3068

cor2$regionIPL -0.9309 0.4151 -2.2424 0.0249 -1.7446 -0.1173 *

cor2$regionmRNFL -0.0276 0.3804 -0.0725 0.9422 -0.7732 0.7181

cor2$regionpRNFL 0.1898 0.3688 0.5145 0.6069 -0.5332 0.9127

cor2$regionretina -0.1530 0.3928 -0.3894 0.6970 -0.9228 0.6169

cor2$regionRPE -0.1591 0.4017 -0.3960 0.6921 -0.9463 0.6282

cor2$sideleft -0.0068 0.2465 -0.0275 0.9780 -0.4899 0.4763

cor2$sideright 0.0715 0.2319 0.3081 0.7580 -0.3831 0.5261

---

Signif. codes: 0 ‘***’ 0.001 ‘**’ 0.01 ‘*’ 0.05 ‘.’ 0.1 ‘ ’ 1

>
