# Supplementary material for: The association between retina thinning and hippocampal atrophy in Alzheimer’s disease and mild cognitive impairment: a meta-analysis and systematic review
Source: Front Aging Neurosci. 2023 Aug 23;15:1232941. doi: 10.3389/fnagi.2023.1232941 (PMC10481874; doi:10.3389/fnagi.2023.1232941)
Supplement: Supplementary file 4 [file Table_4.DOCX]

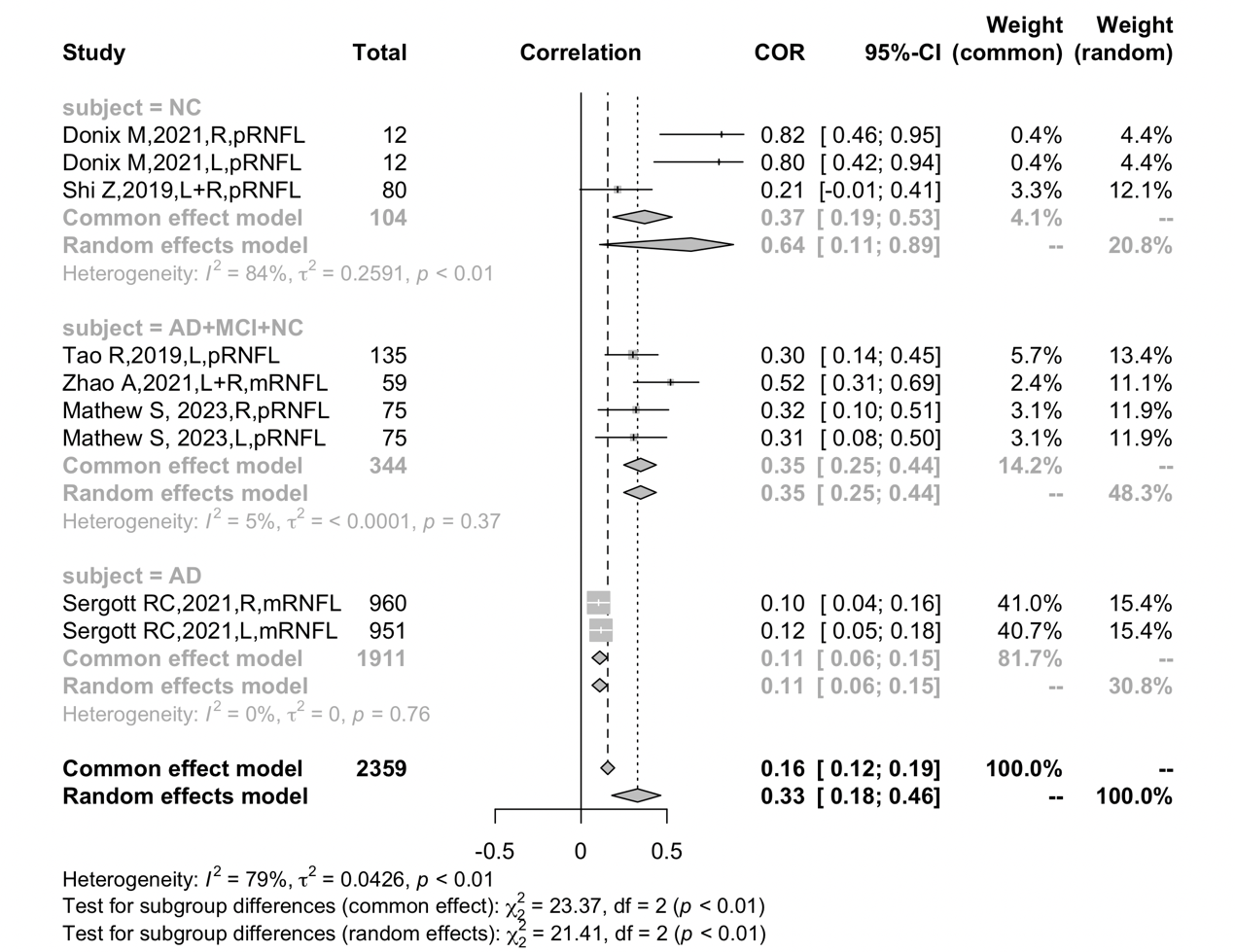


COR 95%-CI %W(common) %W(random) subject

Donix M,2021,R,pRNFL 0.8180 [ 0.4601; 0.9472] 0.4 4.4 NC

Donix M,2021,L,pRNFL 0.8030 [ 0.4249; 0.9425] 0.4 4.4 NC

Tao R,2019,L,pRNFL 0.3020 [ 0.1402; 0.4481] 5.7 13.4 AD+MCI+NC

Sergott RC,2021,R,mRNFL 0.1020 [ 0.0390; 0.1642] 41.0 15.4 AD

Sergott RC,2021,L,mRNFL 0.1160 [ 0.0528; 0.1783] 40.7 15.4 AD

Shi Z,2019,L+R,pRNFL 0.2130 [-0.0070; 0.4134] 3.3 12.1 NC

Zhao A,2021,L+R,mRNFL 0.5210 [ 0.3057; 0.6856] 2.4 11.1 AD+MCI+NC

Mathew S, 2023,R,pRNFL 0.3200 [ 0.1003; 0.5099] 3.1 11.9 AD+MCI+NC

Mathew S, 2023,L,pRNFL 0.3060 [ 0.0849; 0.4984] 3.1 11.9 AD+MCI+NC

Number of studies: k = 9

Number of observations: o = 2359

COR 95%-CI z p-value

Common effect model 0.1555 [0.1156; 0.1948] 7.57 < 0.0001

Random effects model 0.3291 [0.1792; 0.4641] 4.17 < 0.0001

Quantifying heterogeneity:

tau^2 = 0.0426 [0.0163; 0.5080]; tau = 0.2064 [0.1275; 0.7127]

I^2 = 79.4% [61.4%; 89.0%]; H = 2.20 [1.61; 3.01]

Test of heterogeneity:

Q d.f. p-value

38.78 8 < 0.0001

Results for subgroups (common effect model):

k COR 95%-CI Q I^2

subject = NC 3 0.3707 [0.1859; 0.5301] 12.16 83.6%

subject = AD+MCI+NC 4 0.3469 [0.2490; 0.4377] 3.16 5.0%

subject = AD 2 0.1090 [0.0644; 0.1531] 0.10 0.0%

Test for subgroup differences (common effect model):

Q d.f. p-value

Between groups 23.37 2 < 0.0001

Within groups 15.41 6 0.0173

Results for subgroups (random effects model):

k COR 95%-CI tau^2 tau

subject = NC 3 0.6407 [0.1084; 0.8875] 0.2591 0.5091

subject = AD+MCI+NC 4 0.3469 [0.2489; 0.4378] <0.0001 0.0022

subject = AD 2 0.1090 [0.0644; 0.1531] 0 0

Test for subgroup differences (random effects model):

Q d.f. p-value

Between groups 21.41 2 < 0.0001

Details on meta-analytical method:

- Inverse variance method

- Restricted maximum-likelihood estimator for tau^2

- Q-Profile method for confidence interval of tau^2 and tau

- Fisher's z transformation of correlations
